# A conceptual framework for a sports knee injury performance profile (SKIPP) and return to activity criteria (RTAC)

**DOI:** 10.1590/bjpt-rbf.2014.0116

**Published:** 2015-10-06

**Authors:** David Logerstedt, Amelia Arundale, Andrew Lynch, Lynn Snyder-Mackler

**Affiliations:** 1 Department of Physical Therapy, University of the Sciences, Philadelphia, PA, USA; 2 Interdisciplinary Program in Biomechanics and Movement Science, University of Delaware, Newark, DE, USA; 3 Department of Physical Therapy, University of Pittsburgh, Pittsburgh, PA, USA; 4 Center for Sports Medicine, University of Pittsburgh Medical Center, Pittsburgh, PA, USA; 5 Department of Physical Therapy, University of Delaware, Newark, DE, USA

**Keywords:** lower extremity, limb symmetry, sports readiness, athletes

## Abstract

Injuries to the knee, including intra-articular fractures, ligamentous ruptures, and meniscal and articular cartilage lesions, are commonplace within sports. Despite advancements in surgical techniques and enhanced rehabilitation, athletes returning to cutting, pivoting, and jumping sports after a knee injury are at greater risk of sustaining a second injury. The clinical utility of objective criteria presents a decision-making challenge to ensure athletes are fully rehabilitated and safe to return to sport. A system centered on specific indicators that can be used to develop a comprehensive profile to monitor rehabilitation progression and to establish return to activity criteria is recommended to clear athletes to begin a progressive and systematic approach to activities and sports. Integration of a sports knee injury performance profile with return to activity criteria can guide clinicians in facilitating an athlete's safe return to sport, prevention of subsequent injury, and life-long knee joint health.

## Introduction

The burden of musculoskeletal (MSK) injuries on the health of our population is substantial as more than 110 million adults reported musculoskeletal injuries in 2008^1^. MSK injuries are the leading cause of disability in the Unites States with annual direct and indirect costs totaling $950 billion^2^. MSK injuries can be the result of trauma, overuse, or a combination of acute on chronic injury leading to impaired function and reduced quality of life.

The knee is one of the most frequently injured joints in physically active individuals^3^^-^ 5. Many of these injuries, such as intra-articular fractures, ligamentous ruptures, and meniscal and articular cartilage injuries^6^, are traumatic in nature and occur during sports involving jumping, cutting, and pivoting^7^. Surgery for such knee injuries is common, totaling 984,607 arthroscopic knee surgeries performed in the US alone in 2006^8^.

Traumatic knee injuries increase risk for the development of post-traumatic osteoarthritis (PTOA). 

Individuals with a previous knee injury have a 56.8% lifetime risk of development of knee osteoarthritis (OA)^9^, resulting in activity limitations and participation restrictions. Furthermore, 13-18% of patients with total joint replacement report an identifiable traumatic injury to the joint^10^. Brown et al.^11^ estimated that 5.6 million individuals in the United States have PTOA, resulting in annual costs of $3.06 billion. Despite the short-term and long-term risks, many athletes desire to return to cutting and pivoting sports, which increases the risk of additional injuries.

Safe return to sports after a traumatic injury is the responsibility of all healthcare professionals involved. Despite best efforts, athletes returning to high-risk activity and demanding sports after a knee injury are at greater risk of sustaining a second injury. Many post-surgical rehabilitation guidelines are based solely on time from surgery and permit individuals to return to sports-specific activities between 4-9 months; however, very few guidelines provide any objective criteria for assessing an athlete's readiness^12^. The lack of clear objective criteria measuring patient function in sport-specific activities, and for returning to sports may place the injured athlete at risk for re-injury or suboptimal performance. Objective criteria are critical to ensure that athletes are fully rehabilitated and their knees are ready to meet the demands of their sport. Recovery of full function, return to prior activities, and long-term joint health are all goals of the athlete, surgeon, and physical therapist; yet there is little consensus to guide clinicians in facilitating an athlete's safe return to sport, prevention of subsequent injury, and life-long knee joint health.

Currently, there is no system centered on specific indicators that can be used to develop a comprehensive profile to monitor rehabilitation progression and to compile all individualized data to standardize education about the risks of re-injury to the knee and the likelihood of returning to sports. The utilization of these profiles may provide a more accurate and complete representation of an athlete's current status. The purpose of this paper is to build on the conceptual framework for the restoration of limb-to-limb symmetry in its role of secondary and tertiary knee injury prevention by 1) reviewing the epidemiology related to traumatic knee injuries, 2) identifying the risk factors that are associated with re-injury and poor knee function, 3) providing recommendations for objective measures utilizing limb-to-limb symmetry as a performance-based criteria for readiness to return to activity.

## Epidemiology of traumatic knee injuries

### Prevalence

While it is difficult to quantify the number of anterior cruciate ligament (ACL) injuries, recent estimates in the US have reported 81 per 100,000 individuals between the ages of 10 and 64 or about 250,000 per year^13^^-^ 15 with over 127,000 arthroscopic ACL reconstructions (ACLR)^8^. ACL surgeries account for 12.9% of all arthroscopic knee surgeries^8^. ACL injuries often are not isolated; 43-70% of those undergoing ACLR have meniscal lesions, 20-25% have cartilage lesions (about 5% full-thickness) and over 80% have bone bruises^16^^-^ 18.

Meniscal injuries are the fourth most common knee injury in high school athletes^19^. In 2006, medial and lateral meniscal surgeries were the first and third most common arthroscopic surgeries, respectively^8^. In a six-year study encompassing approximately 9% of the US population under the age of 65, there were 387,833 meniscectomies, 23,640 meniscal repairs, and 84,927 ACLR with associated meniscal surgery. Over the six-year time frame, the number of meniscectomies decreased in favor of meniscal repairs^20^, a trend recommended by literature due to the impact on OA. Similar to ACL injuries, meniscal injuries are not common in isolation^18^.

Almost one million individuals are affected annually by articular cartilage injuries^21^^,^ 22. The prevalence of cartilage lesions in the general population is estimated between 5-11%, however in recreational and professional athletes the prevalence is 35%^23^ and higher in athletes participating in cutting and pivoting sports^21^^,^ 22. Upwards of 50% of adolescent athletes participating in cutting and pivoting sports undergoing knee surgery have articular cartilage injuries^24^, and when considering all patients undergoing knee arthroscopy, the prevalence is between 60-70%^25^^-^ 28. Small asymptomatic lesions left untreated can increase in size, resulting in a painful knee joint^29^. Thirty-two to 58% of articular cartilage lesions are the result of a traumatic, noncontact mechanism of injury^25^^,^ 29 ^,^ 30, and as might be expected, nearly three-quarters are concomitant with ACL injuries^17^^,^ 18. Articular cartilage damage after traumatic knee injuries increases the risk of cartilage degradation in all three knee compartments^24^. Consequently, articular cartilage damage is a strong risk factor for the development of osteoarthritis after knee surgeries^31^^,^ 32.

### Failure/Re-injury

Overall, the risk of ACL injury in an athlete with a history of ACLR is 15 times greater than that of a healthy athlete^33^, with an incidence of injury to either the contralateral or ipsilateral knee between 3 and 49%^33^^,^ 34. Athletes with allografts are five times more likely to require a revision compared to those with autografts^35^. There is no significant difference in second injuries between athletes with hamstring autografts and bone-patella-tendon-bone (BPTB) autografts; however, at 15-year follow-up, there were more ipsilateral injuries in the hamstring group and more contralateral injuries in the BPTB group^36^. Returning to cutting/pivoting sports increases the odds of ipsilateral injury 3.9 fold and contralateral 5 fold^37^. Furthermore, positive family history doubles the odds for both ipsilateral and contralateral rupture^37^. Injury side (contralateral vs ipsilateral) is associated with age and graft angle, respectively^38^.

Women with a history of ACL injury are at greater risk of a second ACL injury with 16-fold greater risk of injury compared to healthy controls and four times greater risk than men with a history of ACLR^33^. While most studies have reported an overall greater number of contralateral injuries compared to ipsilateral graft injuries^33^^,^ 38 ^-^ 40, women are six times more likely to suffer a contralateral injury^33^^,^ 40, whereas, men are three times more likely to injure their reconstructed graft^36^.

Younger athletes have greater rates of re-injury within 2 years of ACLR , with 17% of those under the age of 18 having a second ACL injury compared to 7% of those between 18 and 25, and only 4% of those over 25^40^. At three-year follow-up, 29% of those under the age of 20 had a second injury, the highest incidence of any age group^37^. When compared to the older age groups, the youngest age group had a six-fold increase in risk for ipsilateral and three-fold increase for contralateral injury^37^. Leys et al.^36^ calculated an odds ratio of 4.1 for contralateral injury in those under 18. In collegiate athletes, more athletes who had a primary ACLR prior to college went on to have a second injury compared to those who had their primary ACLR during college^41^.

Failure for all meniscal surgeries ranges from 20.2-24.3%, depending on the type of meniscal surgery and status of the ACL^42^. Athletes with meniscal repair and concomitant ACLR have a lower risk of revision for their meniscus injury^43^^-^ 45, suggesting that restoring passive knee stability reduces the incidence of further meniscal damage. Isolated lateral meniscal injury, earlier surgery, older age, and surgeons performing a high volume of meniscal repairs per year also decreases risk of revision^43^^,^ 44. Subsequent operation rates are greater for meniscal repairs compared to partial meniscectomies, greater for partial lateral meniscectomies compared partial medial meniscectomies, and greater for medial meniscus repairs compared to lateral meniscus repairs^46^.

After microfracture, those with a single defect have a lesser failure rate than individuals with multiple defects^47^. Those who had a prior surgery that penetrated the subchondral bone and marrow have a greater failure rate in autologous chondrocyte implantation (ACI) than those who have no history of surgery^48^. In a comparison of individuals who required multiple chondral surgeries, those who received ACI as a first line treatment had lesser failure rates and better International Knee Documentation Committee 2000 Subjective Knee Form (IKDC2000) scores compared to those who had microfracture as their first surgery. Despite a greater failure rate, however, the microfracture group still participated in the same amount of physical activity and at the same frequency and intensity as the ACI group^49^.

### Return to sport

In a recent systematic review of outcomes after ACLR, 88% of athletes returned to sport, 65% returning to their pre-injury level, and 55% returning to competitive play^50^. Athletes who had not returned to sport 12 months after surgery were just as likely to be playing 39 months after surgery as those who had returned to play at 12 months^51^. Self-reported function was different between those playing some sport and those who stopped all activity^52^. Five years after surgery, those who had not returned to sport have worse functional and self-report scores than those who had returned^53^.

Return to sport rates have been reported as high as 98% after meniscal surgery^54^. Even with concomitant grade III or IV articular surface lesions, 48% of individuals in their forties return to sport and 75% resume recreational activities^55^. In athletes under 40, nearly a quarter of those after medial meniscectomies and over half of those after lateral meniscectomies had pain at the time of return to sport; however, pain and swelling were not related to the size of the meniscal resection. In a five-year follow-up study of individuals younger than 45 years old, less than 25% modified their level of athletic participation after partial meniscectomy^55^. However, at 14 years after meniscal surgery, 46% reduced their sporting activity and 6.5% changed occupation as a result of their knees^56^. Seventy-five percent of soccer players after isolated meniscectomy were still playing soccer five years after surgery compared to 52% of those who had combined meniscectomy and ACLR. By the 20-year follow-up, 49% of the isolated meniscectomy group was still playing sports compared to 22% of meniscectomy+ACLR group^57^.

In recreational and amateur athletes, 66% return to sports in eight months after microfracture, with 67% of those eventually returning to a competition level. After 2-5 years however, 49% of athletes have reduced their level of play and 42% have poorer function^58^. In professional sports, return-to-play after microfracture has been studied in the National Football League (NFL) and National Basketball Association (NBA). Seventy-six percent of NFL players^59^ and NBA players^60^^,^ 61 returned to play after microfracture. For most NBA athletes who returned, minutes played per game, points per game, and steals per game decreased compared to pre-surgery. There is conflicting data concerning length of NBA career after microfracture, with some finding no difference^61^ and others finding a decreased likelihood (-LR of 8.15) of continued participation^60^. Average return-to-sport rates in an athletic population after matrix-induced autologous chondrocyte implantation (MACI) is 74%^62^, osteochondral autologous transfer is 91%, and osteochondral allograft transplantation is 88%^62^^,^ 63. ACI is reported to allow the best longevity in sport, with 87% of patients after ACI able to maintain their ability to play at five years after surgery^62^.

### Long-term impact

A history of knee injury, regardless of type, places an individual at a greater risk of subsequent injury. In a study of National Collegiate Athletic Association (NCAA) athletes, those with a history of knee surgery missed more days of sport, had a greater number of knee injuries, and received more magnetic resonance imaging (MRI) tests and surgeries than those athletes with no prior knee injury^64^. Former top-level male athletes with a history of knee injury have a nearly five-fold risk to develop OA^65^. Sports participation and history of ACL injury are both significant risk factors for the development of OA, but meniscal injury in combination with ACL injury may be one of the most potent combinations causing a ten-fold increase in risk compared to age-matched controls^66^^,^ 67.

Total knee arthroplasty and other reconstructive surgeries have advanced significantly in the last decade, allowing former athletes to remain active. However, at a rate of nearly 600,000 per year, with an expected increase to 3.5 million per year by 2030^68^, it is imperative that efforts are made to prevent the need for such surgical procedures.

## Risk factors for re-injury or suboptimal performance upon return to activities

In order to develop a system using rehabilitation indicators for profiling recovery after knee injury or surgery, an understanding of the non-modifiable and modifiable factors that can influence recovery or risk of re-injury is needed. A centralized portal that can track the longitudinal record of rehabilitation indicators can then be used as a means to define an athlete's recovery performance profile. Additionally, profiles can be utilized in establishing criteria to identify thresholds for safe return to sport. Furthermore, the profile can be used as a reference for any rehabilitation specialist interested in developing a similar recovery monitoring system.

### Patient demographics

While patient demographics (e.g. age and sex) are non-modifiable factors, understanding their relationship to re-injury and function can guide clinicians in monitoring and counseling athletes appropriately. Younger athletes with knee injuries typically return more frequently and earlier to sports than older athletes^69^^,^ 70. However, younger athletes (under the age of 25) are also more likely to suffer a second ACL injury after primary ACLR^71^^-^ 73.

Primary knee injury and subsequent 2^nd^ knee injury may be sex-specific. While the incidence of injury to the ACL is greater in men due to the greater exposure to sports, women have a relative risk of injury two to eight times greater than men^74^^,^ 75. However, a recent meta-analysis found no difference between men and women in the risk of patellar tendon graft rupture (Odds ratio (95% confidence interval) = 0.76 (0.29, 2.09)), hamstring graft rupture (Odds ratio = 0.86 (0.53, 1.39)), or contralateral ACL rupture risk (Odds ratio = 0.58 (0.29, 1.17))^76^. Similarly, differences in patient-reported knee function do not appear to be sex-specific^76^, although women may return to less demanding activity levels after ACLR^77^^,^ 78.

### Physical impairments

Range of motion (ROM) symmetry is unique to the individual; however, a knee extension loss of as little as 3^o^ is associated with poor post-surgical, patient-reported outcomes and task-specific activities^79^^-^ 81. Knee ROM asymmetries are also associated with degenerative joint changes^79^^,^ 81.

Muscle strength deficits are pervasive after knee injury and surgery^82^^-^ 85. Muscle strength limb-to-limb symmetry has been proposed as an important marker for readiness to return to unrestricted sport^86^^-^ 89. Early after knee injury, specifically ACL injury, quadriceps strength deficits range from 12-15%^90^^,^ 91. Pre-operative quadriceps strength deficits are predictive of poor functional outcomes after ACLR^92^^-^ 94. The largest extent of quadriceps weakness occurs in the first six months after knee surgery^92^^,^ 95 ^,^ 96 and can be as great as 39-40%^84^^,^ 97 ^-^ 102. While hamstring strength deficits may be present after knee injury or surgery, these deficits do not influence clinical or functional outcomes^103^^-^ 105. However, the hamstrings-to-quadriceps ratio for torque production has been reported as a factor in primary ACL injury risk model^106^^,^ 107.

Quadriceps strength deficits can persist for months or years after any knee joint surgery, in spite of rehabilitation^84^^,^ 108. Consistent deficits in quadriceps strength have been found after surgery for the ACL, meniscus, and articular cartilage within the first year^109^, 2 years^110^^,^ 111, and up to 7 years^112^^-^ 114. Side-to-side deficits of more than 10% to 15% are considered significant and should be assessed throughout rehabilitation, and even into the second and third post-operative seasons^109^^-^ 114.

Quadriceps strength asymmetry can also be reflected in other impairment measures. Quadriceps index (QI) is expressed as a percentage of the peak value of the quadriceps muscles on the involved side divided by the peak value of the quadriceps muscles on the uninvolved side. After ACLR, those with QI less than 85% have worse hop scores than those with a QI greater than 90% or controls. QI is a better predictor of hop test distance than graft type, presence of meniscal injury, knee pain, or knee symptoms^115^. After meniscectomy, particularly in middle-aged athletes, greater quadriceps strength is associated with better self-reported knee joint function on all five subscales of the Knee Injury and Osteoarthritis Outcome Score (KOOS)^116^. The KOOS is a knee-specific, patient-reported instrument for knee injuries that can lead to post-traumatic osteoarthritis. The form includes 42 items in five separately scored subscales: Pain (9 items); other symptoms (7 items); function in activities of daily living (ADLs; 17 items); function in sport and recreation (Sports; 5 items); and knee-related quality of life (QoL; 4 items)^117^. Individuals, two years after meniscectomy, continued to have a mean 6% asymmetry in strength and scored between 10 and 26 points worse on all five KOOS subscales compared to controls^118^.

Balance and postural deficits have been reported after knee injury, and in particular, after ACL injury and reconstruction. Various assessments have been used to evaluate risk of injury, current status, and the magnitude of improvement after an intervention^119^. While static postural tasks may provide useful clinical information, dynamic postural tasks may provide a more accurate representation of sporting activities. Some of these tasks may be simple, such as the Star Excursion Balance Test and Y-Balance tests^119^, while others require instrumented equipment^120^^,^ 121. Though some authors have tried quantifying limb symmetry for postural deficits, evidence is limited^122^.

### Performance-based measures

Performance-based measures can be used to assess a combination of muscle strength, neuromuscular control, confidence in the injured limb, and ability to complete sport-specific activities^123^. Many drills and performance-based measures are double-legged tasks; however, the performance may mask persistent deficits in the injured lower extremity^124^. Therefore, single-legged tasks should be used after knee injuries to detect side-to-side differences, evaluate function, monitor progress of rehabilitation, and assess readiness for return to sports^123^^,^ 125 ^-^ 128. Single-legged hop tests measure distance, time, or height and typically involve multi-movement patterns (i.e. multi-planar directions, change of direction, acceleration-deceleration, etc.) that attempt to resemble athletic movements and may prepare patients for return to sporting activities^129^^-^ 132.

Side-to-side limb symmetry appears to have a critical role in the prevention of injury and return to sports after knee injuries. Varying performance standards (i.e. muscle strength or hop performance), ranging from 70% to 90% limb symmetry index (LSI), have been suggested as benchmarks for determining normal symmetry^86^^,^ 125 ^,^ 132 ^-^ 134. However, this range provides health care professionals no indication of an expected standard or a timeline on which they should be achieved.

Early after injury or surgery, individuals have poor single-legged hop LSI and substantial limb-to-limb differences^83^^,^ 123 ^,^ 129. Performance deficits on single-legged hop tests range from 5-35% with up to 47% of athletes not achieving normal limb symmetry (85-90% LSI) six months after surgery^83^^,^ 85 ^,^ 129 ^,^ 135. By 12 months, the average LSI is greater than 90%, and by 24 months, individuals are able to maintain normal hop symmetry^83^^,^ 85 ^,^ 136. LSI calculated from the cross-over hop for distance and 6-meter timed single-legged hop tests can also predict self-reported knee function at one year after ACLR. Poor LSI can predict poor knee function, while normal LSI can predict normal knee function^137^^,^ 138. Athletes six months after ACLR with an LSI less than 88% for the 6-meter timed hop were five times more likely to rate themselves below normal ranges on the IKDC2000 one year after ACLR, whereas athletes with an LSI greater than 95% on the cross-over hop six months after ACLR were four times more likely to rate themselves within normal ranges on the IKDC2000 one year after ACLR^138^. Despite improvements in single-legged hop performance and symmetry in the first year after ACLR^83^^,^ 129, athletes two years after surgery have greater asymmetries in single-legged hop distances when compared to controls^139^. Poor LSI and large limb-to-limb differences prior to seven months after ACLR reconstruction can be a concern, as most post-surgical rehabilitation guidelines enable individuals to return to sports-specific activities between 4 to 6 months^140^^,^ 141. It is likely that sports-specific activities are more challenging than landing from a planned hop in a controlled environment, thus the deficits seen in single-legged hop performance may be magnified, potentially predisposing the ipsilateral or contralateral knee to injury. Because hop testing assesses current knee function, individuals with poor LSI may exhibit suboptimal performance on the playing field and may be placed at greater risk for injury^88^^,^ 142 ^,^ 143.

When comparing individuals after ACI and after microfracture, those after ACI have greater single hop asymmetry than those after microfracture six and twelve months after surgery. However, there is no difference between the groups in cross-over or 6-meter timed hop tests at six and twelve months. At 24 months, the microfracture groups had minimal asymmetry in hop performance (4-8% asymmetry), while the ACI group had larger asymmetries (10-17% asymmetry) on all three hop tests^111^ .

### Symptoms

Persistent symptoms, such as knee pain, joint swelling, stiffness, instability, or weakness, are common reasons many athletes cite for not returning to preinjury activity levels^69^. While pain may be a potential indicator of incomplete healing^144^, it typically resolves after knee injury and/or surgery. One year after ACLR, athletes who had not returned to sports reported an average pain intensity of 1.0±1.1 out of 10 (0=no pain, 10=worst imaginable pain)^145^. Upon returning to sport after meniscectomy, pain and effusion can persist and should be monitored^70^. Pain can have a role in the decision-making process for allowing athletes to safely return to sports, but it should not be the sole determinant. Pain and effusion can be reliably monitored using a pain-monitoring scale^146^, soreness guidelines^147^, and the modified stroke test^148^.

Joint effusion is an over-accumulation of fluid within the joint capsule, indicating inflammation or irritation^148^. Joint effusion can be helpful in establishing a diagnosis, determining exercise progression, and monitoring progress. The presence of effusion can impair adjacent muscle function and alter knee motion^149^^,^ 150. The presence of no effusion is also a significant contributor for the likelihood of return to sports one year after ACLR^145^. Monitoring of joint effusion can be practically, reliability, and clinically useful. The modified stroke test and effusion grading scale offers an objective means of measuring and assessing knee joint effusion^148^. This modified stroke test is performed by sweeping fluid proximally out of the medial sulcus of the knee, and then performing a distally directed sweep along the lateral knee and watching for a wave of fluid returning to the medial sulcus^148^. An increase in effusion following treatment that does not return to baseline likely indicates that treatment progression was too aggressive. Furthermore, individuals should be able to demonstrate the ability to tolerate lower loading demands without pain or swelling before progressing to higher loads.

Symptomatic knee joint instability (giving way) is a hallmark of knee joint injury. Giving way episodes are usually described as buckling at the knee similar to the initial injury. While the magnitude of passive ligament instability is poorly associated with functional ability in ACL-deficient athletes^151^^-^ 153, dynamic knee stability may be more relevant. Subsequently, the absence of episodes of knee instability was a significant contributor (Wilks' λ=0.357) for the likelihood of return to sports one year after ACLR^145^. Recurrent episodes of instability may be an indicator of undiagnosed concomitant injuries (other ligamentous structures, meniscus) and can potentially increase the likelihood of further joint damage^25^.

### Gait asymmetry

While the measurement of movement using motion capture is not considered a typical rehabilitation indicator, it does provide additional insight on the ubiquity of asymmetries seen after knee injury and surgery. Side-to-side asymmetrical movement patterns after knee injury are common and can persist for months or years after injury or surgery^154^. Additionally, these altered movement patterns are not limited to the index knee. Neuromuscular adaptations are present in the hip and ankle and contralateral limb after knee injury and surgery as well as during gait and higher-demand tasks, such as jumping^154^^-^ 157. Underlying neuromuscular imbalances on the operated and non-operated limbs at the time of return to sport clearance are highly predictive of 2^nd^ ACL injury^158^.

Even two years after ACLR, Roewer et al.^108^ found that the involved limb had smaller knee excursion and internal knee extension moments compared to the uninvolved limb at weight acceptance. Gait asymmetries after ACLR are associated with poor quadriceps strength and functional performance. At peak knee flexion, those with a QI less than 90% have a smaller knee flexion angle and significantly decreased internal knee extension moment compared to controls^159^. QI alone accounts for more than a quarter of variance in angle at peak knee flexion, and QI and KOS-ADLS accounts for 60% of variance in internal knee extension moment^159^. Broadening the criteria to include QI, single-legged hop LSI, KOS-ADLS, and GRS, those who scored less than 90% on any one of these measures had greater knee kinematic and kinetic asymmetries than those who scored greater than 90% on all criteria and had clinically significant limb-to-limb asymmetry in hip flexion at peak knee flexion^160^.

In higher-risk tasks such as jumping, women two years after ACLR demonstrate limb-to-limb asymmetries. Higher vertical ground reaction force and loading rate is seen on the uninvolved limb during landing compared to both the involved limb and controls^161^. During takeoff, women also show lower force generation on the involved side compared to the uninvolved side^161^. Both men and women after ACLR have smaller internal knee extension moments on the involved limb during lateral step-down and vertical jump take-off and landing when compared to the uninvolved limb and to controls^162^. The results of these studies and similar research highlight the need to resolve impairments and restore functional limb symmetry after ACLR.

Gait asymmetries have been noted following meniscal surgery. Smaller peak knee flexion angles and lower peak external moments in the sagittal plane and larger knee adduction moments have been observed in the involved limb compared to the uninvolved limb after partial meniscectomy and compared to controls^163^. Asymmetry may be worse in individuals with weaker quadriceps after partial meniscectomy. Increased average and peak external knee adduction moments throughout stance phase have been observed in patients after meniscectomy with weaker quadriceps compared to those patients after meniscectomy with normal strength and controls^164^. Bulgheroni et al.^165^ found that those after medial meniscectomy had decreased external hip extension moment during all phases of gait and increased external knee flexion moment at loading response, push off, and throughout swing. They also had increased hip and knee flexion and increased ankle dorsiflexion in late swing phase^165^.

After ACI surgery, aberrant movement patterns are present, specifically reduced knee motion during weight acceptance and decreased external sagittal plane moments. These aberrant patterns can persist for months^157^^,^ 166, and alter joint loading^167^^-^ 169. Gait deviations may promote further cartilage damage through reduced shock absorption and increased joint loading^170^^,^ 171, predisposing the knee to degenerative changes^167^^,^ 172.

### Patient-reported outcomes

Patient-reported outcome (PRO) measures are self-report questionnaires that measure an individual's perception of daily life and physical activity^173^^,^ 174. PROs show a greater relationship to on patient satisfaction than standard clinical measures^175^. PROs specific to the knee joint contain items to assess symptoms (i.e. pain, swelling, giving ways, etc.) and activity limitations (i.e. ambulation, stair climbing, running, etc.)^176^. PROs are clinically useful in comparing the results of interventions on patient perspective after injury^175^^,^ 177. Performance-based measures capture different domains of function than PROs^125^^,^ 178. Performance-based measures assess the actual functional ability of an athlete, whereas, PROs assess the perceived ability of aspects considered important by patients with knee problems, ranging from stair climbing to running and jumping. Therefore, a combination of outcome measures is likely necessary to provide a comprehensive evaluation of functional success^178^^,^ 179.

Knee performance and self-reported function generally improve over the first year after ACLR^83^. By six months after surgery, almost half of individuals score greater than 90% on Knee Outcome Survey-Activities of Daily Living Scale (KOS-ADLS) and Global Rating Scale of Perceived Function (GRS), and 78% have achieved these scores by 12 months^180^. Poor self-report on outcome measures after ACLR are associated with chondral injury, previous surgery, return to sport, and poor radiological grade in ipsilateral medial compartment^181^. ACLR revision and extension deficits at 3 months are also predictors of poor long-term, patient-reported outcomes^17^^,^ 182.

The various surgical techniques for articular cartilage defects vary in their self-reported outcomes. Pooled data indicates that 67% of individuals report normal IKDC2000 one year after microfracture, and 80% of individuals have significant increases from pre-surgery in Lysholm, Tegner, and KOOS sports subscale scores^183^. While these patients have made significant improvements in their self-reported function, a large proportion of them continue to report function below normal levels. Four years after microfracture, the KOOS-ADL subscale, Marx Activity Rating Scale, and Tegner Score decreased in 47% of athletes, but despite this decrease in self-report, 44% were still able to regularly participate in pivoting sports, and 57% of those at their pre-operative level^184^. In a 15-year longitudinal study, IKDC2000, Lysholm, and Tegner scores decreased over the course of the study; however, they were still better at 15 years after surgery than at baseline before surgery^185^. After osteochondral allograft transplantation for large chondral or osteochondral defects, athletes who returned to sport had better IKDC2000, KOOS, and Marx Activity Rating Scale scores^186^. Kreuz et al.^187^ compared inactive/rarely active individuals to active individuals after autologous chondrocyte implantation (ACI). Pre-operatively, there were no differences between groups, but at 6, 12, and 36 months after surgery, the active group had significantly better International Cartilage Repair Society (ICRS) and Cincinnati Knee Rating System scores compared to the inactive/rarely active group^187^. All five KOOS subscales increase over the course of the first one to two years after MACI and improvements are maintained five years after surgery. Improvements in IKDC2000, modified Cincinnati Knee Rating System, Tegner, Lysholm, and Short Form-36 (SF36) scores as well as knee extension range of motion continue to gradually improve over the first five years following MACI^188^^,^ 189.

## Decision-making considerations and the importance of symmetry

Determining when a patient is performing well enough to safely and effectively return to play or activity is a complex decision that must take into account the risk factors for re-injury or poor function. Meeuwisse et al.^190^ has proposed a model that integrates the risk of the athlete within a dynamic sporting environment, considering both intrinsic and extrinsic factors that lead to different injury events and their variability. The remainder of this commentary outlines a proactive decision-making model that measures changes in intrinsic and extrinsic factors and can be predictive, preventative, personalized, and participatory. This model can provide rehabilitation specialists crucial data pertinent to patients' current knee function, their progress during rehabilitation, the necessity for additional rehabilitation, and their readiness to return to sporting activities.

Creighton et al.^144^ has proposed a decision-based return-to-play model that involves three steps. Step 1 is an evaluation of health status that focuses on type and severity of the injury, clinical or physical signs and symptoms, functional performance, and psychological state. Step 2 evaluates the risk of sport participation from the type of sport, competition level, or position played to the use of protective equipment. Step 3 or decision-modification step frequently involves nonmedical factors, such as timing and season of the sport, external pressures to compete, and legal implications. For this review, we will focus on the components highlighted in Step 1 (Evaluation of Health Status) as these are likely the medical/clinical risk factors that can be modified by the clinician. The medical/clinical variables that are frequently associated with return to activities or sports include demographic factors, physical impairments, activity limitations, psychological factors, and patient-reported scores145,191,192.

Limb-to-limb symmetry or limb symmetry indexes are used after injury or surgery as important indicators of physical impairments, activity limitations, and function. They are also used to monitor the progress of rehabilitation and to assess readiness for return to activity or sports, therefore they should be benchmarked against performance standards. Unfortunately, no empirically based benchmarks or expert consensus benchmarks exist regarding performance standards at specific time points after knee injury. Development of standards can provide relevant information about patient performance and can help to determine if additional interventions are needed to achieve this level.

## Profiling and monitoring recovery of athletes after knee injury or surgery

Injury to the meniscus, cartilage, or ligaments of the knee results in a fairly consistent clinical presentation and an increase in the risk for post-traumatic osteoarthritis. Creating a profile of these individuals is important to promote a comprehensive evaluation of the patient to eliminate basic impairments after surgery and to facilitate a safe and effective return to sport process that minimizes the risk for second injury. Additionally, using a consistent set of measures at consistent time frames allows for assessment of trends in patient outcomes.

One of the challenges in developing an injury risk profile for post-injury or post-operative management has been to select appropriate clinical or field tests that can detect side-to-side asymmetries, assess global knee function, and determine a patient's readiness to return to sport. Batteries of tests have been developed to predict the risk for musculoskeletal injuries^193^, classify individuals early after ACL injury^194^, and identify important limb asymmetries after ACL injury and reconstruction^195^^,^ 196. One battery of performance-based tests was moderately correlated with the IKDC2000 and could discriminate between the operated and non-operated limbs of patients after ACLR^197^. However, very few studies incorporate performance-based and patient-reported outcomes into the clinical decision making to fully evaluate a patient's knee function^12^^,^ 198. Clinical impairments, performance-based measures, and patient-reported outcomes capture different aspects of overall knee performance and are important indicators of function^125^^,^ 199 ^-^ 201. Therefore, a battery of tests utilizing performance-based and patient-reported outcomes can provide clinically relevant data applicable to current knee function, progress throughout rehabilitation and the necessity for additional targeted interventions, and their readiness to return to sporting activities.

### Sports knee injury performance profile

While many different tests and measures are available for functional testing^202^^,^ 203, the Sports Knee Injury Performance Profile (SKIPP) is a battery of tests and measures consisting of thigh muscle strength testing, single-legged hop testing, and patient-reported outcome measures. The data included in the SKIPP have not been independently validated; however, that process is ongoing. Prior to performing the battery of tests, athletes should exhibit a minimum criteria of little to no joint effusion, full active range of motion, normal gait pattern upon visual observation, and ability to hop in place on a single leg without pain.

Quadriceps and hamstring strength can be tested using isokinetic peak torque or maximal voluntary isometric contraction (MVIC). Peak force or torque values achieved during strength testing bilaterally are recorded and used to calculate a quadriceps index (QI) or hamstrings index (HI). QI is expressed as a percentage of the peak value of the quadriceps muscles on the involved side divided by the peak value of the quadriceps muscles on the uninvolved side. Hamstrings index is expressed similarly.

Following quadriceps strength testing, participants perform single-legged hop tests. Four single-legged hop tests are used in our clinic: single hop for distance (single hop); cross-over hop for distance (cross-over hop); triple hop for distance (triple hop); and 6-meter timed hop^132^. A hop score for each test is calculated as the average of the two recorded trials. For the single hop, cross-over hop, and triple hop LSI, these LSIs are expressed as the percentage performance on the involved side compared to the uninvolved side. For the 6-meter timed hop, the 6-meter timed hop LSI is expressed as the percentage performance of the uninvolved side compared to the involved side, given that faster times (low numbers) are better for this hop test.

Following hop testing, participants complete self-report questionnaires: KOS-ADLS, GRS, IKDC2000, and the ACL-Return to Sports after Injury (ACL-RSI). The KOS-ADLS is a 14-item patient-reported outcome of symptoms and functional limitations of the knee during ADLs^204^. Patients must be able to perform their ADLs at a normal level prior to attempting a return to sports, otherwise they are likely to report having difficulty with sporting activities and placing themselves at risk for subpar performance and re-injury. The GRS asks participants to rate their current knee function on a scale from 0 to 100, with 0 being the inability to perform any activity and 100 being the level of knee function prior to the injury, including sports^204^^,^ 205. The IKDC2000 is a frequently used assessment of function^206^ and can differentiate between individuals with low versus high knee function^207^. The published IKDC2000 normative dataset^207^ provides a reference standard for normal knee function (Table 1)^137^^,^ 138. 

**Table 1.** IKDC2000 cutoff scores for normal ranges for age- and sex-specific groups

**Age Group**	**Normal IKDC2000 cutoff**
18-24	Men: 89.7 Women: 83.9
25-34	Men: 86.2 Women: 82.8
35-50	Men: 85.1 Women: 78.5
51-65	Men: 74.7 Women: 69.0

ACL-RSI is a 12-item patient-reported outcome of emotions, confidence in performance, and risk appraisal after ACLR. It can discriminate psychological differences between athletes who returned to sports and those who did not return to sports^208^^,^ 209. The data collected from this battery of tests can be used as a set of performance indicators that can detect side-to-side asymmetries, assess global knee function, and determine a patient's readiness to return to sport. This permits the clinician to visualize and appreciate the dynamic profile of the injured athlete and aids the clinician in decision making about readiness to return to activity and in the formulation of targeted, personalized interventions to overcome performance barriers and optimize sports performance.

### Recommendations for return to activity criteria

The rehabilitation indicators from the SKIPP can be used to determine readiness to return to activities or sports - an improvement over the current timebased rehabilitation protocols. No functional test battery for return to sports has been validated to identify cutoffs which reduce the risk of injury to this point. Despite impairments, activity restrictions, poor self-report scores, and limb-to-limb asymmetries, many post-surgical rehabilitation guidelines permit individuals to return to sports-specific activities between three to nine months after surgery, depending on the lesion and surgical technique. However, the use of a time-based approach does not adequately account for these deficits^210^. A majority of clinicians continue to use a time-based approach and passive stability measures to allow return to play after ACLR^211^. A recent systematic review noted that only one or two criteria (muscle strength and single-leg hop test) have been used as objective measures for resuming play in the majority of studies^12^. In studies that did use objective measurable criteria, none provided cutoffs for their criteria that have been validated for normal knee function, successful return to activities, or re-injury rates^12^^,^ 211 ^,^ 212. Objective, measurable criteria are critical to ensure that athletes are fully rehabilitated and their knees are ready to meet the demands of their activities or sport.

Recently, two paradigms of return to activity criteria have been proposed by the European Board of Sports Rehabilitation and the University of Delaware as recommendations to clear athletes to begin a progressive and systematic approach to activities and sports^89^^,^ 194. The European Board of Sports Rehabilitation have developed a set of criteria using performance-based measures prior to athletes returning to activities or sport after ACLR^89^. Their recommendations are categorized based on type of activity: activities that are pivoting, contact, or competitive and activities that are non-pivoting, non-contact, or recreational. For the pivoting/contact/competitive group, they recommend that involved limb knee extensor and knee flexor muscle strength performance be equal to 100% of the uninvolved limb (100% LSI) and that involved limb hop performance on two maximum hop tests 

(e.g. single hop for distance, vertical hop, etc.) and one endurable hop test (e.g. triple hop, stair hop, side hop, etc.) be at least 90% of the uninvolved limb (90% LSI). For the non-pivoting/non-contact/recreational group, they recommend that involved limb knee extensor and knee flexor muscle strength performance be at least 90% of the uninvolved limb (90% LSI) and that involved limb hop performance on one maximum or one endurable hop tests be at least 90% of the uninvolved limb (90% LSI). These recommendations take into account both knee extensor and knee flexor strength and hop performance; however, one limitation of these recommendations is the omission of the use of PROs as criteria for return to activity. As stated before, PROs do not correlate highly with performance-based measures, but capture different aspects of knee function. It has been suggested that both performance-based measures and PROs are needed to fully characterize an athlete's knee function^83^.

**Figure 1.**
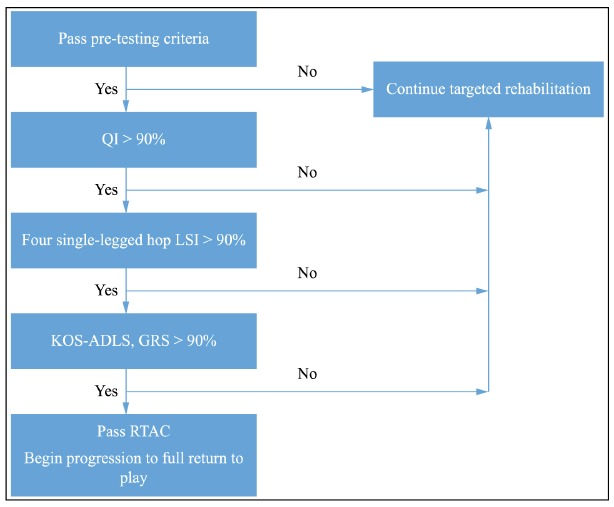
Algorithm for passing return to activity criteria.

The University of Delaware has instituted return to activity criteria and used them for over 15 years^194^. Functional testing to determine return to activity criteria includes performance-based and PRO measures from the SKIPP. These criteria are sensitive to knee functional changes over time and can provide clinicians with clinically relevant information about patients' responses to different therapeutic interventions^83^. To pass return to activity, participants were required to achieve 90% or greater on each of the functional tests and measures from the battery of tests (QI, 4 hop LSIs, KOS-ADLS, and GRS) (Figure 1)^86^^,^ 194. 

Work from our laboratory has demonstrated that the University of Delaware Return to Activity Criteria (RTAC) can accurately discern between two differently functioning cohorts of athletes after ACL injury or reconstruction. The RTAC demonstrated that participants who successfully returned to high-level activity after non-operative management of an ACL injury had less than a 10% deficit on their baseline scores on average^194^. Athletes who fail our RTAC six months after ACLR exhibit greater limb-to-limb movement asymmetries than those who pass our RTAC^69^. Six and 12 months after ACL surgery, poor IKDC2000 function scores were reasonably indicative of RTAC test battery failure, whereas normal IKDC2000 scores were not predictive of passing scores on the RTAC test battery^138^. Additionally, those athletes who demonstrated limb-to-limb movement symmetry and self-reported knee function 6 months after ACLR are more likely to return to their preinjury activity level 12 months after ACLR^213^. The results of these studies highlight the importance of using performance-based and patient-reported measures to identify participants with poor knee function and limb-to-limb movement asymmetry before clearing them to return to high-demand activities. The use of these RTAC can be used early after any knee injury or surgery to assess residual deficits that needed to be resolved prior to attempting a return to high-risk sporting activities.

The validation of the RTAC to determine safe and optimal return to activities or to predict future injuries is ongoing. Several clinical variables have been identified with returning to sports^145^^,^ 191 ^,^ 192 ^,^ 198; however, none have been studied to predict future injury^154^^,^ 214. Further research is needed to identify if the tests in the SKIPP and the criteria for the RTAC can accurately identify which athletes are more likely to return to activities and which ones are more likely to sustain a second injury.

## Conclusions

Knee injuries are common in sports. Despite the advances made in surgical techniques and rehabilitation interventions, return to sport rates are poorer than previously thought, and the risk of re-injury or failure after knee surgery is greater than expected. The development of the Sports Knee Injury Performance Profile allows the clinician to consistently monitor knee function, track progress throughout rehabilitation, and incorporate targeted, personalized interventions to achieve optimal sports performance and function while potentially reducing the risk of re-injury or failure. The implementation of established return to activity criteria provides a platform to ensure that athletes are fully rehabilitated and can begin to introduce loads needed to participate in their sport or activities. Consistent implementation of this profile will allow clinicians to track individual patient progress and to assess trends in their patients over time.
